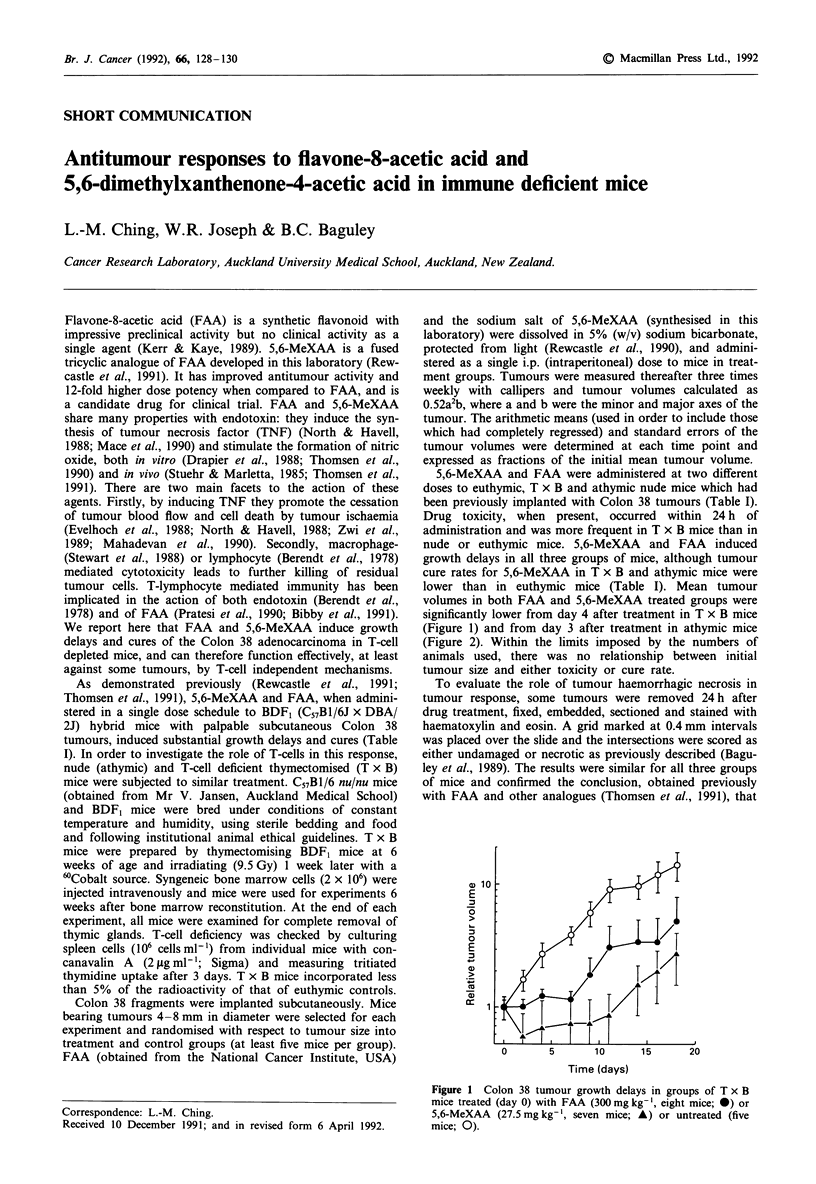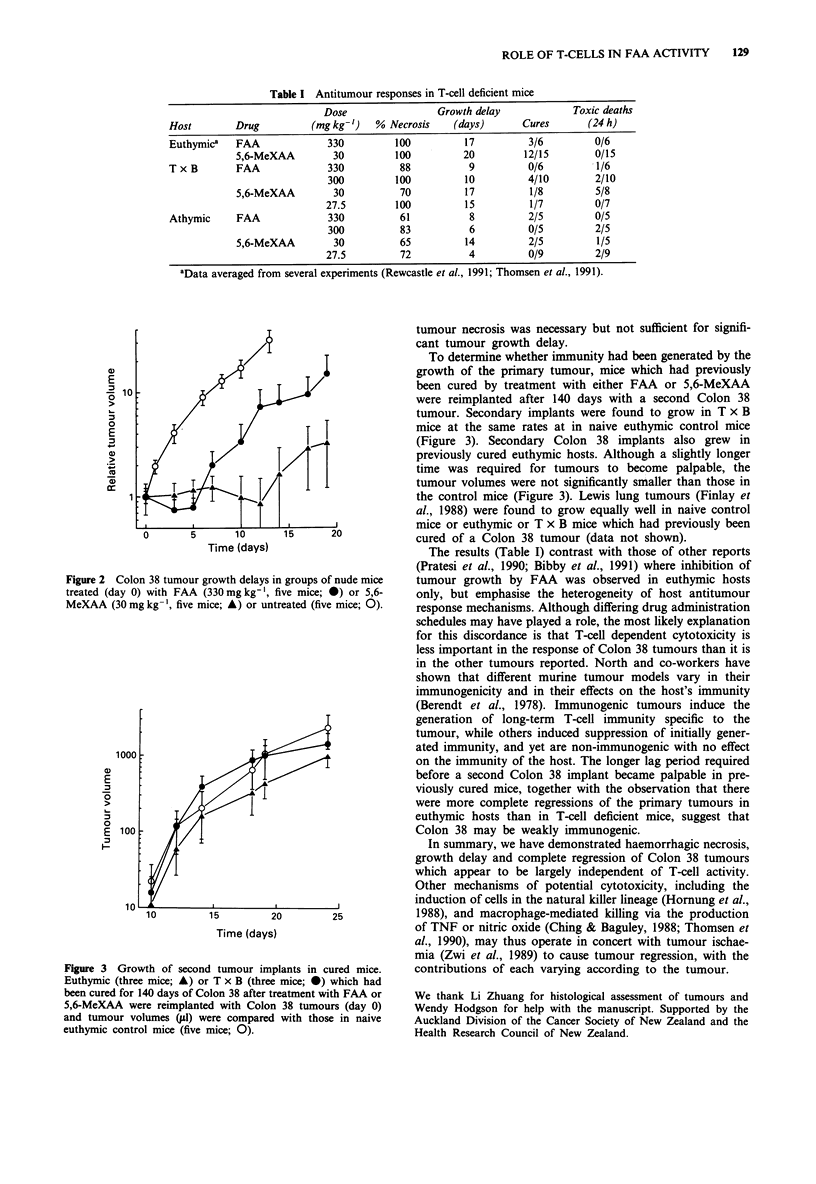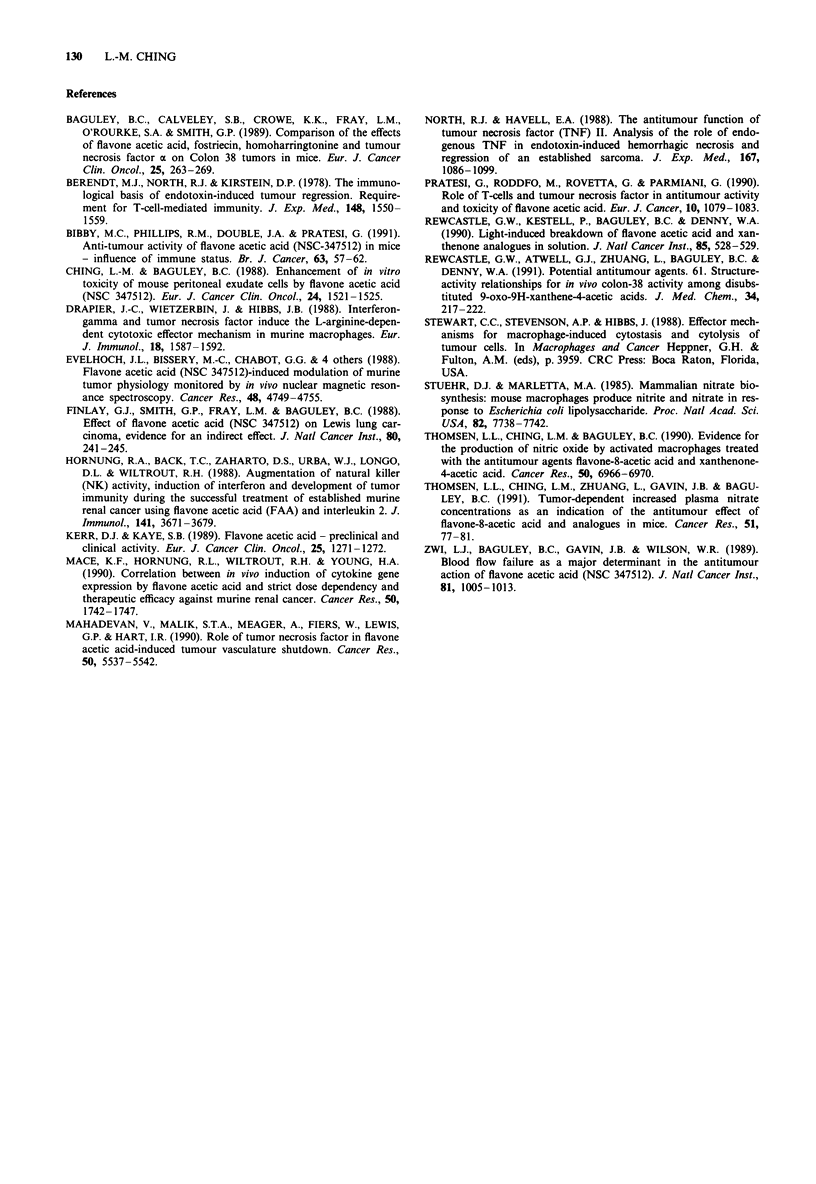# Antitumour responses to flavone-8-acetic acid and 5,6-dimethylxanthenone-4-acetic acid in immune deficient mice.

**DOI:** 10.1038/bjc.1992.228

**Published:** 1992-07

**Authors:** L. M. Ching, W. R. Joseph, B. C. Baguley

**Affiliations:** Cancer Research Laboratory, Auckland University Medical School, New Zealand.


*Br. J. Cancer* (1992), **66**, 128–130 © Macmillan Press Ltd., 1992

SHORT COMMUNICATION

### Antitumour responses to flavone-8-acetic acid and 5,6-dimethylxanthenone-4-acetic acid in immune deficient mice

L.-M. Ching, W.R. Joseph & B.C. Baguley

*Cancer Research Laboratory, Auckland University Medical School, Auckland, New Zealand.*

Flavone-8-acetic acid (FAA) is a synthetic flavonoid with impressive preclinical activity but no clinical activity as a single agent (Kerr & Kaye, 1989). 5,6-MeXAA is a fused tricyclic analogue of FAA developed in this laboratory (Rewcastle *et al.*, 1991). It has improved antitumour activity and 12-fold higher dose potency when compared to FAA, and is a candidate drug for clinical trial. FAA and 5,6-MeXAA share many properties with endotoxin: they induce the synthesis of tumour necrosis factor (TNF) (North & Havell, 1988; Mace *et al.*, 1990) and stimulate the formation of nitric oxide, both *in vitro* (Drapier *et al.*, 1988; Thomsen *et al.*, 1990) and *in vivo* (Stuehr & Marletta, 1985; Thomsen *et al.*, 1991). There are two main facets to the action of these agents. Firstly, by inducing TNF they promote the cessation of tumour blood flow and cell death by tumour ischaemia (Evelhoch *et al.*, 1988; North & Havell, 1988; Zwi *et al.*, 1989; Mahadevan *et al.*, 1990). Secondly, macrophage- (Stewart *et al.*, 1988) or lymphocyte (Berendt *et al.*, 1978) mediated cytotoxicity leads to further killing of residual tumour cells. T-lymphocyte mediated immunity has been implicated in the action of both endotoxin (Berendt *et al.*, 1978) and of FAA (Pratesi *et al.*, 1990; Bibby *et al.*, 1991). We report here that FAA and 5,6-MeXAA induce growth delays and cures of the Colon 38 adenocarcinoma in T-cell depleted mice, and can therefore function effectively, at least against some tumours, by T-cell independent mechanisms.

As demonstrated previously (Rewcastle *et al.*, 1991; Thomsen *et al.*, 1991), 5,6-MeXAA and FAA, when administered in a single dose schedule to $BDF_1$ ($C_{57}Bl/6J \times DBA/2J$) hybrid mice with palpable subcutaneous Colon 38 tumours, induced substantial growth delays and cures (Table I). In order to investigate the role of T-cells in this response, nude (athymic) and T-cell deficient thymectomised (T × B) mice were subjected to similar treatment. $C_{57}Bl/6$ *nu/nu* mice (obtained from Mr V. Jansen, Auckland Medical School) and $BDF_1$ mice were bred under conditions of constant temperature and humidity, using sterile bedding and food and following institutional animal ethical guidelines. T × B mice were prepared by thymectomising $BDF_1$ mice at 6 weeks of age and irradiating (9.5 Gy) 1 week later with a $^{60}$Cobalt source. Syngeneic bone marrow cells ($2 \times 10^6$) were injected intravenously and mice were used for experiments 6 weeks after bone marrow reconstitution. At the end of each experiment, all mice were examined for complete removal of thymic glands. T-cell deficiency was checked by culturing spleen cells ($10^6$ cells ml$^{-1}$) from individual mice with concanavalin A (2 $\mu$g ml$^{-1}$; Sigma) and measuring tritiated thymidine uptake after 3 days. T × B mice incorporated less than 5% of the radioactivity of that of euthymic controls.

Colon 38 fragments were implanted subcutaneously. Mice bearing tumours 4–8 mm in diameter were selected for each experiment and randomised with respect to tumour size into treatment and control groups (at least five mice per group). FAA (obtained from the National Cancer Institute, USA) and the sodium salt of 5,6-MeXAA (synthesised in this laboratory) were dissolved in 5% (w/v) sodium bicarbonate, protected from light (Rewcastle *et al.*, 1990), and administered as a single i.p. (intraperitoneal) dose to mice in treatment groups. Tumours were measured thereafter three times weekly with callipers and tumour volumes calculated as $0.52a^2b$, where a and b were the minor and major axes of the tumour. The arithmetic means (used in order to include those which had completely regressed) and standard errors of the tumour volumes were determined at each time point and expressed as fractions of the initial mean tumour volume.

5,6-MeXAA and FAA were administered at two different doses to euthymic, T × B and athymic nude mice which had been previously implanted with Colon 38 tumours (Table I). Drug toxicity, when present, occurred within 24 h of administration and was more frequent in T × B mice than in nude or euthymic mice. 5,6-MeXAA and FAA induced growth delays in all three groups of mice, although tumour cure rates for 5,6-MeXAA in T × B and athymic mice were lower than in euthymic mice (Table I). Mean tumour volumes in both FAA and 5,6-MeXAA treated groups were significantly lower from day 4 after treatment in T × B mice (Figure 1) and from day 3 after treatment in athymic mice (Figure 2). Within the limits imposed by the numbers of animals used, there was no relationship between initial tumour size and either toxicity or cure rate.

To evaluate the role of tumour haemorrhagic necrosis in tumour response, some tumours were removed 24 h after drug treatment, fixed, embedded, sectioned and stained with haematoxylin and eosin. A grid marked at 0.4 mm intervals was placed over the slide and the intersections were scored as either undamaged or necrotic as previously described (Baguley *et al.*, 1989). The results were similar for all three groups of mice and confirmed the conclusion, obtained previously with FAA and other analogues (Thomsen *et al.*, 1991), that

**Figure 1** Colon 38 tumour growth delays in groups of T × B mice treated (day 0) with FAA (300 mg kg$^{-1}$, eight mice; ●) or 5,6-MeXAA (27.5 mg kg$^{-1}$, seven mice; ▲) or untreated (five mice; ○).

Correspondence: L.-M. Ching.
Received 10 December 1991; and in revised form 6 April 1992.

**Table I** Antitumour responses in T-cell deficient mice

| *Host* | *Drug* | *Dose (mg kg⁻¹)* | *% Necrosis* | *Growth delay (days)* | *Cures* | *Toxic deaths (24 h)* |
|---|---|---|---|---|---|---|
| Euthymic[a] | FAA | 330 | 100 | 17 | 3/6 | 0/6 |
| | 5,6-MeXAA | 30 | 100 | 20 | 12/15 | 0/15 |
| T x B | FAA | 330 | 88 | 9 | 0/6 | 1/6 |
| | | 300 | 100 | 10 | 4/10 | 2/10 |
| | 5,6-MeXAA | 30 | 70 | 17 | 1/8 | 5/8 |
| | | 27.5 | 100 | 15 | 1/7 | 0/7 |
| Athymic | FAA | 330 | 61 | 8 | 2/5 | 0/5 |
| | | 300 | 83 | 6 | 0/5 | 2/5 |
| | 5,6-MeXAA | 30 | 65 | 14 | 2/5 | 1/5 |
| | | 27.5 | 72 | 4 | 0/9 | 2/9 |

[a]Data averaged from several experiments (Rewcastle *et al.*, 1991; Thomsen *et al.*, 1991).

**Figure 2** Colon 38 tumour growth delays in groups of nude mice treated (day 0) with FAA (330 mg kg$^{-1}$, five mice; ●) or 5,6-MeXAA (30 mg kg$^{-1}$, five mice; ▲) or untreated (five mice; ○).

**Figure 3** Growth of second tumour implants in cured mice. Euthymic (three mice; ▲) or T x B (three mice; ●) which had been cured for 140 days of Colon 38 after treatment with FAA or 5,6-MeXAA were reimplanted with Colon 38 tumours (day 0) and tumour volumes (μl) were compared with those in naive euthymic control mice (five mice; ○).

tumour necrosis was necessary but not sufficient for significant tumour growth delay.

To determine whether immunity had been generated by the growth of the primary tumour, mice which had previously been cured by treatment with either FAA or 5,6-MeXAA were reimplanted after 140 days with a second Colon 38 tumour. Secondary implants were found to grow in T x B mice at the same rates at in naive euthymic control mice (Figure 3). Secondary Colon 38 implants also grew in previously cured euthymic hosts. Although a slightly longer time was required for tumours to become palpable, the tumour volumes were not significantly smaller than those in the control mice (Figure 3). Lewis lung tumours (Finlay *et al.*, 1988) were found to grow equally well in naive control mice or euthymic or T x B mice which had previously been cured of a Colon 38 tumour (data not shown).

The results (Table I) contrast with those of other reports (Pratesi *et al.*, 1990; Bibby *et al.*, 1991) where inhibition of tumour growth by FAA was observed in euthymic hosts only, but emphasise the heterogeneity of host antitumour response mechanisms. Although differing drug administration schedules may have played a role, the most likely explanation for this discordance is that T-cell dependent cytotoxicity is less important in the response of Colon 38 tumours than it is in the other tumours reported. North and co-workers have shown that different murine tumour models vary in their immunogenicity and in their effects on the host's immunity (Berendt *et al.*, 1978). Immunogenic tumours induce the generation of long-term T-cell immunity specific to the tumour, while others induced suppression of initially generated immunity, and yet are non-immunogenic with no effect on the immunity of the host. The longer lag period required before a second Colon 38 implant became palpable in previously cured mice, together with the observation that there were more complete regressions of the primary tumours in euthymic hosts than in T-cell deficient mice, suggest that Colon 38 may be weakly immunogenic.

In summary, we have demonstrated haemorrhagic necrosis, growth delay and complete regression of Colon 38 tumours which appear to be largely independent of T-cell activity. Other mechanisms of potential cytotoxicity, including the induction of cells in the natural killer lineage (Hornung *et al.*, 1988), and macrophage-mediated killing via the production of TNF or nitric oxide (Ching & Baguley, 1988; Thomsen *et al.*, 1990), may thus operate in concert with tumour ischaemia (Zwi *et al.*, 1989) to cause tumour regression, with the contributions of each varying according to the tumour.

We thank Li Zhuang for histological assessment of tumours and Wendy Hodgson for help with the manuscript. Supported by the Auckland Division of the Cancer Society of New Zealand and the Health Research Council of New Zealand.

### References

BAGULEY, B.C., CALVELEY, S.B., CROWE, K.K., FRAY, L.M., O'ROURKE, S.A. & SMITH, G.P. (1989). Comparison of the effects of flavone acetic acid, fostriecin, homoharringtonine and tumour necrosis factor α on Colon 38 tumors in mice. *Eur. J. Cancer Clin. Oncol.*, **25**, 263-269.

BERENDT, M.J., NORTH, R.J. & KIRSTEIN, D.P. (1978). The immunological basis of endotoxin-induced tumour regression. Requirement for T-cell-mediated immunity. *J. Exp. Med.*, **148**, 1550-1559.

BIBBY, M.C., PHILLIPS, R.M., DOUBLE, J.A. & PRATESI, G. (1991). Anti-tumour activity of flavone acetic acid (NSC-347512) in mice - influence of immune status. *Br. J. Cancer*, **63**, 57-62.

CHING, L.-M. & BAGULEY, B.C. (1988). Enhancement of *in vitro* toxicity of mouse peritoneal exudate cells by flavone acetic acid (NSC 347512). *Eur. J. Cancer Clin. Oncol.*, **24**, 1521-1525.

DRAPIER, J.-C., WIETZERBIN, J. & HIBBS, J.B. (1988). Interferon-gamma and tumor necrosis factor induce the L-arginine-dependent cytotoxic effector mechanism in murine macrophages. *Eur. J. Immunol.*, **18**, 1587-1592.

EVELHOCH, J.L., BISSERY, M.-C., CHABOT, G.G. & 4 others (1988). Flavone acetic acid (NSC 347512)-induced modulation of murine tumor physiology monitored by *in vivo* nuclear magnetic resonance spectroscopy. *Cancer Res.*, **48**, 4749-4755.

FINLAY, G.J., SMITH, G.P., FRAY, L.M. & BAGULEY, B.C. (1988). Effect of flavone acetic acid (NSC 347512) on Lewis lung carcinoma, evidence for an indirect effect. *J. Natl Cancer Inst.*, **80**, 241-245.

HORNUNG, R.A., BACK, T.C., ZAHARTO, D.S., URBA, W.J., LONGO, D.L. & WILTROUT, R.H. (1988). Augmentation of natural killer (NK) activity, induction of interferon and development of tumor immunity during the successful treatment of established murine renal cancer using flavone acetic acid (FAA) and interleukin 2. *J. Immunol.*, **141**, 3671-3679.

KERR, D.J. & KAYE, S.B. (1989). Flavone acetic acid - preclinical and clinical activity. *Eur. J. Cancer Clin. Oncol.*, **25**, 1271-1272.

MACE, K.F., HORNUNG, R.L., WILTROUT, R.H. & YOUNG, H.A. (1990). Correlation between *in vivo* induction of cytokine gene expression by flavone acetic acid and strict dose dependency and therapeutic efficacy against murine renal cancer. *Cancer Res.*, **50**, 1742-1747.

MAHADEVAN, V., MALIK, S.T.A., MEAGER, A., FIERS, W., LEWIS, G.P. & HART, I.R. (1990). Role of tumor necrosis factor in flavone acetic acid-induced tumour vasculature shutdown. *Cancer Res.*, **50**, 5537-5542.

NORTH, R.J. & HAVELL, E.A. (1988). The antitumour function of tumour necrosis factor (TNF) II. Analysis of the role of endogenous TNF in endotoxin-induced hemorrhagic necrosis and regression of an established sarcoma. *J. Exp. Med.*, **167**, 1086-1099.

PRATESI, G., RODDFO, M., ROVETTA, G. & PARMIANI, G. (1990). Role of T-cells and tumour necrosis factor in antitumour activity and toxicity of flavone acetic acid. *Eur. J. Cancer*, **10**, 1079-1083.

REWCASTLE, G.W., KESTELL, P., BAGULEY, B.C. & DENNY, W.A. (1990). Light-induced breakdown of flavone acetic acid and xanthenone analogues in solution. *J. Natl Cancer Inst.*, **85**, 528-529.

REWCASTLE, G.W., ATWELL, G.J., ZHUANG, L., BAGULEY, B.C. & DENNY, W.A. (1991). Potential antitumour agents. 61. Structure-activity relationships for *in vivo* colon-38 activity among disubstituted 9-oxo-9H-xanthene-4-acetic acids. *J. Med. Chem.*, **34**, 217-222.

STEWART, C.C., STEVENSON, A.P. & HIBBS, J. (1988). Effector mechanisms for macrophage-induced cytostasis and cytolysis of tumour cells. In *Macrophages and Cancer* Heppner, G.H. & Fulton, A.M. (eds), p. 3959. CRC Press: Boca Raton, Florida, USA.

STUEHR, D.J. & MARLETTA, M.A. (1985). Mammalian nitrate biosynthesis: mouse macrophages produce nitrite and nitrate in response to *Escherichia coli* lipolysaccharide. *Proc. Natl Acad. Sci. USA*, **82**, 7738-7742.

THOMSEN, L.L., CHING, L.M. & BAGULEY, B.C. (1990). Evidence for the production of nitric oxide by activated macrophages treated with the antitumour agents flavone-8-acetic acid and xanthenone-4-acetic acid. *Cancer Res.*, **50**, 6966-6970.

THOMSEN, L.L., CHING, L.M., ZHUANG, L., GAVIN, J.B. & BAGULEY, B.C. (1991). Tumor-dependent increased plasma nitrate concentrations as an indication of the antitumour effect of flavone-8-acetic acid and analogues in mice. *Cancer Res.*, **51**, 77-81.

ZWI, L.J., BAGULEY, B.C., GAVIN, J.B. & WILSON, W.R. (1989). Blood flow failure as a major determinant in the antitumour action of flavone acetic acid (NSC 347512). *J. Natl Cancer Inst.*, **81**, 1005-1013.